# Machine learning discovery of longitudinal patterns of depression and suicidal ideation

**DOI:** 10.1371/journal.pone.0222665

**Published:** 2019-09-20

**Authors:** Jue Gong, Gregory E. Simon, Shan Liu

**Affiliations:** 1 Department of Industrial and Systems Engineering, University of Washington, Seattle, WA, United States of America; 2 Kaiser Permanente Washington Health Research Institute, Seattle, WA, United States of America; 3 Psychiatry and Behavioral Sciences, University of Washington, Seattle, WA, United States of America; University of Toronto, CANADA

## Abstract

**Background and aim:**

Depression is often accompanied by thoughts of self-harm, which are a strong predictor of subsequent suicide attempt and suicide death. Few empirical data are available regarding the temporal correlation between depression symptoms and suicidal ideation. We investigated the anecdotal concern that suicidal ideation may increase during a period of depression improvement.

**Data:**

Longitudinal Patient Health Questionnaire (PHQ)-9 is a questionnaire of 9 multiple-choice questions to assess the frequency of depressive symptoms within the previous two weeks. We analyzed a chronic depression treatment population’s electronic health record (EHR) data, containing 610 patients’ longitudinal PHQ-9 scores (62% age 45 and older; 68% female) within 40 weeks.

**Methods:**

The irregular and sparse EHR data were transformed into continuous trajectories using Gaussian process regression. We first estimated the correlations between the symptoms (total score of the first 8 questions; PHQ-8) and suicide ideation (9th question score; Item 9) using the cross-correlation function. We then used an artificial neural network (ANN) to discover subtypes of depression patterns from the fitted depression trajectories. In addition, we conducted a separate analysis using the unfitted raw PHQ scores to examine PHQ-8’s and Item 9’s pattern changes.

**Results:**

Results showed that the majority of patients’ PHQ-8 and Item 9 scores displayed strong temporal correlations. We found five patterns in the PHQ-8 and the Item 9 trajectories. We also found 8% - 13% of the patients have experienced an increase in suicidal ideation during the improvement of their PHQ-8. Using a trajectory-based method for subtype pattern detection in depression progression, we provided a better understanding of temporal correlations between depression symptoms over time.

## Introduction

Depression is a complex and dynamic mental disorder that is among the leading causes of disability worldwide [1]. In the United States, an estimated 7% of all adults had at least one major depressive episode in year 2015 [2]. A common practice in the prevention and treatment of chronic depression is to identify risk signals for serious health complications; these signals include various types of longitudinal predictors such as severity of depression symptoms and suicidal ideation. Depression is often accompanied by thoughts of self-harm, which are believed to be a predictor of subsequent suicide attempt and suicide death. Simon et al. [3,4] found that the risk of a suicide attempt increases with a higher frequency of thoughts on self-harm. A meta-analysis of longitudinal studies on suicide risk factors showed that the most common outcome (48%) was suicide attempt after self-injurious thoughts and behavior, but the effect was considerably weaker than anticipated [5]. Franklin et al. [6] pointed out that traditional methods on identifying risk factors of self-injurious thoughts are limited, and highlighted the need for machine learning-based algorithm to examine suicidal ideation risk.

Furthermore, Mittal et al. [7] claimed that improvement in depression may be accompanied by an increase in suicidal ideation. Few empirical studies are available regarding the temporal association between depression symptoms and suicidal ideation. Evidence collection and interpretation are further complicated by the heterogeneity in patients’ depression symptom trajectories. We define trajectories as longitudinal records of symptom severities that form progression patterns. Additionally, most available data regarding temporal associations between suicidal ideation and other symptoms of depression are derived from clinical trials rather than patients treated in community practices [7–9].

This study focuses on the temporal correlation between depression and suicidal ideation for a heterogeneous patient population, in which we assume there are patterns in the depression progression and suicide ideation trajectories. The objective of this study is to discover heterogeneous patterns of depression trajectories and investigate the concern that suicidal ideation may increase during a period of depression improvement by using the electronic health record (EHR) data. This study does not try to predict suicide death or suicide attempts from depression severity measures using the EHR.

Depression severity is measured by the Patient Health Questionnaire (PHQ)-9; a self-administered questionnaire that includes 9 multiple-choice questions to assess the frequency of depressive symptoms within the previous two weeks [10]. The total score without the 9th question, the PHQ-8 score, has similar ability to predict major depressive disorder compared to the PHQ-9 score [10]. The 9th question (Item 9), which asks about suicidal ideation, is analyzed as a separate symptom in this study. Previous research has shown that Item 9 identifies patients at increased risk of suicide attempt [4]. Mittal et al. [7] questioned the belief in the clinical community that patients with depression are at increased risk of suicide as they begin to recover and their motivation returns. They found such claim remains to be substantiated. The relationship between depression improvement and change in suicidal ideation has not been supported by analysis of depression and suicidal ideation trajectory data.

EHRs containing depression records of a large number of individuals over extended periods can be useful in discovering heterogeneity in depression trajectories. However, common statistical methods on modeling trajectories often fail to accommodate the statistical challenges of longitudinal EHR data, such as data sparsity and irregularity [11,12]. Research on modeling depression trajectories based on longitudinal datasets have emerged since 2005 [13]. There are wide variations in the time intervals between consecutive symptom observations, with most studies reporting six months or longer. The long gap in observations is problematic because depression assessment questionnaires such as the PHQ-9 asks patients to report symptoms in the past two weeks. Therefore, sparse measurements may not represent the true underlying trajectories [9].

Common methods for discovering unknown patterns in longitudinal trajectories include unsupervised learning method such as k-means clustering [14], hierarchical clustering [15], and more sophisticated group-based trajectory models that build individual growth curves and then cluster them to subgroups based on learned model parameters [16]. These group-based trajectory models assume the population is consisted of multiple homogenous or heterogeneous groups, and trajectory parameters are estimated for each group or individuals within each group [13]. Major methods include Growth Mixture Model (GMM), Generalized Growth Mixture Model (GGMM), Latent Class Analysis (LCA), Latent Class Growth Analysis (LCGA), Latent Class Growth Mixture Model (LCGMM), and Collaborative Modeling (CM) [17–20]. The majority of these models are based on distributional theory; e.g. individuals as random samples from multivariate distributions. Given the nature of EHR data, we cannot guarantee the theoretical consistency of these methods. Others on this list require advanced optimization capability [20], assumptions on the form of the disease models, and domain knowledge of the number of subgroups.

In this study, we address these data challenges in the EHR by selecting patients with frequent records of observations. We follow the method proposed by Lasko et al. [21] for computational phenotype discovery over noisy, sparse, and irregular EHR data. We apply a data-driven and model-free method (i.e., no assumption on the form of the disease model) based on an artificial neural network (ANN) to extract subtypes of depression with respect to the local progression patterns from collected trajectory information. One benefit of an ANN is its ability to transform longitudinal data of large volume to latent variables as a high-level abstraction of the original data [22]. In addition, our model of latent structure learning discovers patterns in the trajectories without a prior knowledge on the subtypes; this is a form of unsupervised learning. Using the ANN, we reconstruct the depression trajectory of an individual patient by learning the patterns which are a set of latent basis trajectories without the need of building individual statistical models. Next, subtypes of depression are discovered by classifying patients based on these learned latent patterns [23]. Therefore, we can identify trajectories of overall depression severity and trajectories of suicidal ideation and examine their synchrony.

Using the ANN and cross-correlation analysis, this study aims to contribute to the understanding of depression progression by discovering heterogeneous patterns and estimating the temporal correlation between depressive symptoms and suicide ideation trajectories. To the best of our knowledge, this paper provides the first look of trajectories of PHQ-8 and Item 9 for chronic depression patients with ongoing treatment. This work consists of three parts: the first is estimating the temporal correlation between depression symptoms and suicide ideation trajectories; the second is finding evidence that improvement in depression symptoms and increase in suicidal ideation may happen simultaneously in a subset of patients; the third is learning latent patterns and clusters of similar patients from trajectories of depression symptoms in the EHR data.

## Materials and methods

### Data description

The empirical data were provided by four health systems participating in the Mental Health Research Network (HealthPartners, and the Colorado, Washington, and Southern California regions of Kaiser Permanente), totaling 1.2 million observations [3,4,20,24]. All four health systems recommend routine use of the PHQ-9 questionnaire at all specialty mental health visits and at all primary care visits including diagnosis or treatment of depression. The dataset includes individuals’ longitudinal PHQ-9 measures between years 2007 and 2012, and are linked to relative time between measurements, type of providers (primary care, specialist, mental health) where the questionnaire was conducted, individuals’ age, sex, race/ethnicity, diagnosis and treatment status, and the Charlson Comorbidity Index (a common indicator of chronic disease severity).

In the EHR data, 9,306 individuals have received ongoing depression treatment (defined as either receiving antidepressant medication or making specialty mental health visits for depression during the past 6 months). We selected a subset containing 610 patients (62% age 45 and older; 68% female) with at least six PHQ-9 scores recorded during twenty consecutive two-week periods from the original sample. These patients have chronic depression and are relatively similar on their stage of treatment. No patients have initiated new therapies in the past 6 months or longer. The summary statistics of this group is listed in Table A in S1 File. The PHQ-9 records do not have equal intervals between two consecutive data points. Individual scores for each PHQ-9 question are used to compute the PHQ-8 and Item 9 scores. We are interested in the correlation between the trajectory of overall depression severity (as indicated by the PHQ-8 score) and the trajectory of suicidal ideation (as indicated by Item 9 score), which can be described by the cross-correlation function.

This study only included de-identified patients’ EHRs and all analyses have been carried out in accordance with the approved guidelines by institutional review board through Kaiser Permanente Washington Health Research Institute (KPWHRI) and Mental Health Research Network (MHRN). The data that support the findings of this study are available from the MHRN, but restrictions apply to the availability of these data. Data may be available from the authors upon reasonable request and with permission of KPWHRI and MHRN.

### Fitting continuous trajectories

We first transformed the irregular records of each patient’s PHQ-8 and Item 9 scores into continuous trajectories using Gaussian process regression (GPR); we then sampled the data at equal biweekly time interval for further analysis. We followed the method and notation as defined in Lasko et al. [21]. Each time period is two weeks. We used grid-based methods to search for the optimal parameters in the regression with respect to maximizing the mean of marginal likelihood over all patients (details see Appendix B in S1 File). Two sets of parameters were used for the PHQ-8 scores and Item 9.

### Cross-correlation of multiple time series

We investigated the temporal correlations between PHQ-8 and Items 9 score using the cross-correlation function (CCF). CCF is a measure of similarity of two series as a function of a relative displacement (i.e. a lag of *k* time units). For two series *x*_*t*_ and *y*_*t*_ which are observed from stationary stochastic processes, their sample CCF $\hat{\rho }(k)$ is defined as

$${\hat{\rho }}_{xy}(k)=\frac{{\hat{\gamma }}_{xy}(k)}{{\hat{\sigma }}_{x}{\hat{\sigma }}_{y}},$$

where ${\hat{\sigma }}_{x},\;{\hat{\sigma }}_{y}$ are the sample deviations of the processes, ${\hat{\gamma }}_{xy}(k)$ is the sample cross-covariance at lag *k*, defined as

$${\hat{\gamma }}_{xy}(k)=\left\{ \begin{array}{l} \;\frac{1}{N-k}\sum_{t=1}^{N-k}\left(x_{t+k}-\bar{x})(y_{t}-\bar{y}\right),\;k\geq 0\\ \frac{1}{N-|k|}\sum_{t=1}^{N-|k|}\left(x_{t}-\bar{x})(y_{t+|k|}-\bar{y}\right),\;k<0 \end{array}\right.$$

Where *N* is the number of observations; $\bar{x},\;\bar{y}$ are the estimated mean values of the time series [25]. The range of CCF is between -1 to 1, with values close to 1 representing high similarity, values close to 0 representing low similarity, and values close to -1 indicating a negative/inverse relationship between two time series. Incorporating the lag function, for example, if CCF is close to 1 at lag *k*<0, then the shape of series *x*_*t*_ is similar to *y*_*t*_ at a time of |*k*| units ahead, indicating that *x*_*t*_ leads *y*_*t*_ with |*k*| units.

### Analyses of PHQ-8 and Item 9 scores using unfitted observations

To examine the belief that depression improvement and increase in suicidal ideation may happen simultaneously as stated in Mittal et al. [7], we used the Spearman’s rank-order correlation to measure the strength and direction of association between the PHQ-8 and Item 9 changes. We considered both short-term effect from changes in PHQ-8 and Item 9 values between consecutive observations within 1 month, and long-term effects from changes in the two values with longer time windows. For the 610 subjects, we collected the following data for both PHQ-8 and Item 9 using unfitted/raw observations in the EHR data: (1) the rate of change of the PHQ-8 and the Item 9 scores within 1 month (i.e. the difference of the scores between two consecutive observations within 1 month divided by the period length); (2) the first observation (month 0); (3) the average of the records between month *t*– 0.5 and *t* + 0.5 for month *t* = 3, 6, 9. The PHQ-8 scores were converted to the average score of each question with a range of 0 to 3.

Furthermore, we investigated two patterns of PHQ-8 and Item 9 changing in opposite directions using unfitted/raw observations in the EHR dataset: (a) PHQ-8 increases and Item 9 decreases, and (b) PHQ-8 decreases and Item 9 increases. We defined an “Item 9 change” as an increasing or decreasing score by at least 1 unit between observations, and a “PHQ-8 change” as a score increasing or decreasing by at least *d* units between observations, where *d* is the threshold. We tested *d* = 2,3,4 in this study.

### Artificial neural network for latent feature detection

We used autoencoder, a type of artificial neural network [21,26], to discover the latent structure of depression trajectories in a population. Neural network for subtype discovery has been applied on other diseases including gout and leukemia [21]. We assumed that the trajectory of a patient can be decomposed as a linear combination of a set of basis trajectories. We treated these basis trajectories as latent patterns of the population. Each basis trajectory represents for a trend in the depression severity, such as an increasing trend in severity over time, or an increasing and then decreasing trend. The value of the coefficient of each pattern, called the activation, stands for how strongly the basis trajectory is represented in the patient’s trajectory. We further used these activations as features to extract a measure of similarities among patients. The latent structure consists of the latent patterns of basis trajectories and the feature of activation of each patient in the population.

An autoencoder is a special neural network structure with three layers: (1) an input layer of *M* nodes representing the fitted trajectories; (2) a latent layer of *H* nodes representing the hidden features by transforming the input (the encoder); and (3) the output layer with *M* nodes representing a reconstruction of the input data by transforming the latent layer (the decoder) [21,26]. The encoder transforms an input trajectory represented as a vector $m\in R^{M}$ into the latent representation $h\in R^{H}$ by **h** = *u*(**Wm**+**b**), where $W\in R^{H\times M}$ is a matrix of latent features, $b\in R^{H}$ is the bias vector, and *u* is a pre-specified nonlinear function. The decoder computes a reconstruction $\hat{m}$ with the function $\hat{m}=W^{⊤}h+b^{\prime }$. The training of parameters of the autoencoder is described in Appendix B in S1 File. Each row of **W** after training is a vector representing one of the learned patterns or basis trajectories. The **W** forms a compact set of basis trajectories that can be linearly combined to represent the input trajectory. The resulting element *h*_*i*_ is the activation of pattern **W**_*i*_ for the input **m**, indicating how strongly **W**_*i*_ is represented in **m**. The cost function of the autoencoder (see Appendix B in S1 File) was minimized using the stochastic gradient descent method [26]. We used cross-validation to determine the hyperparameters in the model, including the number of latent nodes (*H*). The training samples were randomly split into five subsamples with equal size. A single instance of cross-validation was to use one of the five subsamples as the validation data, and the remaining four subsamples as training data. The cross-validation process was repeated 5 times with each of the five subsamples used exactly once as the validation data. The criterion of validation is the mean squared error (MSE) between the original trajectory and the reconstructed trajectory.

There is a unique activation vector for each patient to represent the similarity of this patient to each basis group. We can cluster the patients using the activation vector to discover the subtypes of depression. We used the k-means clustering algorithm [14]. The number of clusters was decided by comparing the inertia, the sum of squared distances to the closest centroid for all observations (see Appendix D in S1 File).

All analyses was performed using Python. The GPR and the k-means algorithms were implemented with the open source package scikit-learn [27,28]. The neural network was implemented with the package Theano [28].

## Results and discussion

### Cross-correlation between measurements

**Fig 1:**
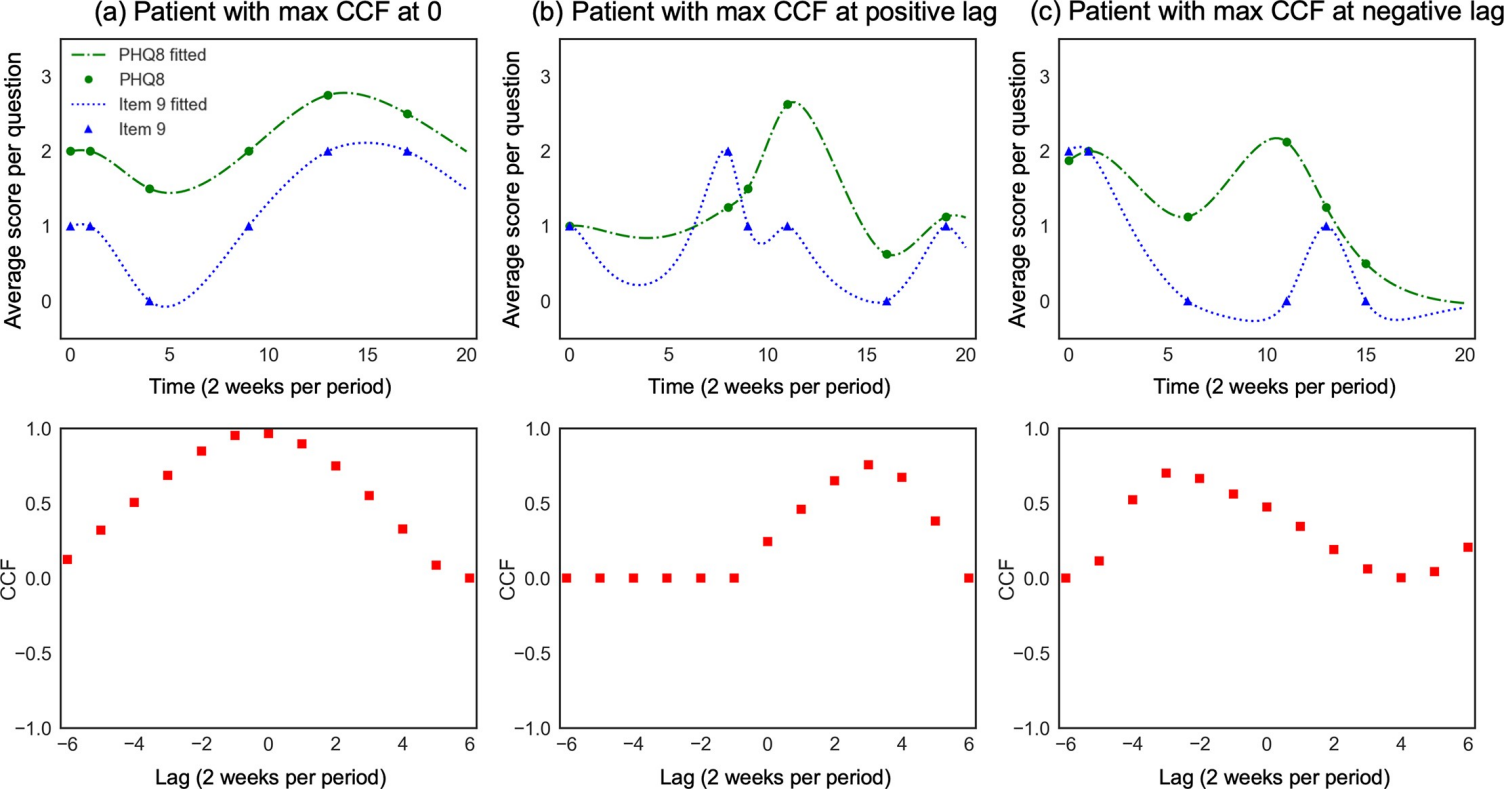
Three sample patients’ CCFs between time series. The first row is the records of the two series (PHQ-8 and Item 9) for each patient and their fitted curve; the second row is the CCFs between PHQ-8 and Item 9. One unit of time is two weeks.

The goal is to examine the temporal similarity between two depressive symptom measurements. We estimated the cross-correlation between the fitted trajectories of PHQ-8 and Item 9 for each patient, sampled with a period of 2 weeks. We provided three examples of patient’s CCF between PHQ-8 and Item 9 in Fig 1. To compare PHQ-8 and Items 9 on the same scale, we used the average score of the first 8 questions in the PHQ to represent PHQ-8. In Fig 1(A), this patient’s CCF between PHQ-8 and Item 9 peaks at lag *k* = 0, which indicates that the two trajectories are most similar with no lag, thus her PHQ-8 and Item 9 follow the same trend at the same time. In Fig 1(B), this patient’s CCF between PHQ-8 and Item 9 peaks at lag *k* = 1; this positive lag means that her PHQ-8 and Item 9 are the most similar if PHQ-8 moves 1 unit to the left; this can be interpreted as her Item 9 and PHQ-8 change with a most similar trend such that Item 9 leads one time period (i.e. 2 weeks). In Fig 1(C), this patient’s CCF between PHQ-8 and Item 9 peaks at lag *k* = −2; this negative lag means that his PHQ-8 and Item 9 are the most similar if PHQ-8 moves two units to the right. We did not observe any negative CCF for lags between -5 and 5, which means there is no strong evidence in this dataset that the processes of depression and suicidal ideation have negative temporal correlation.

**Fig 2:**
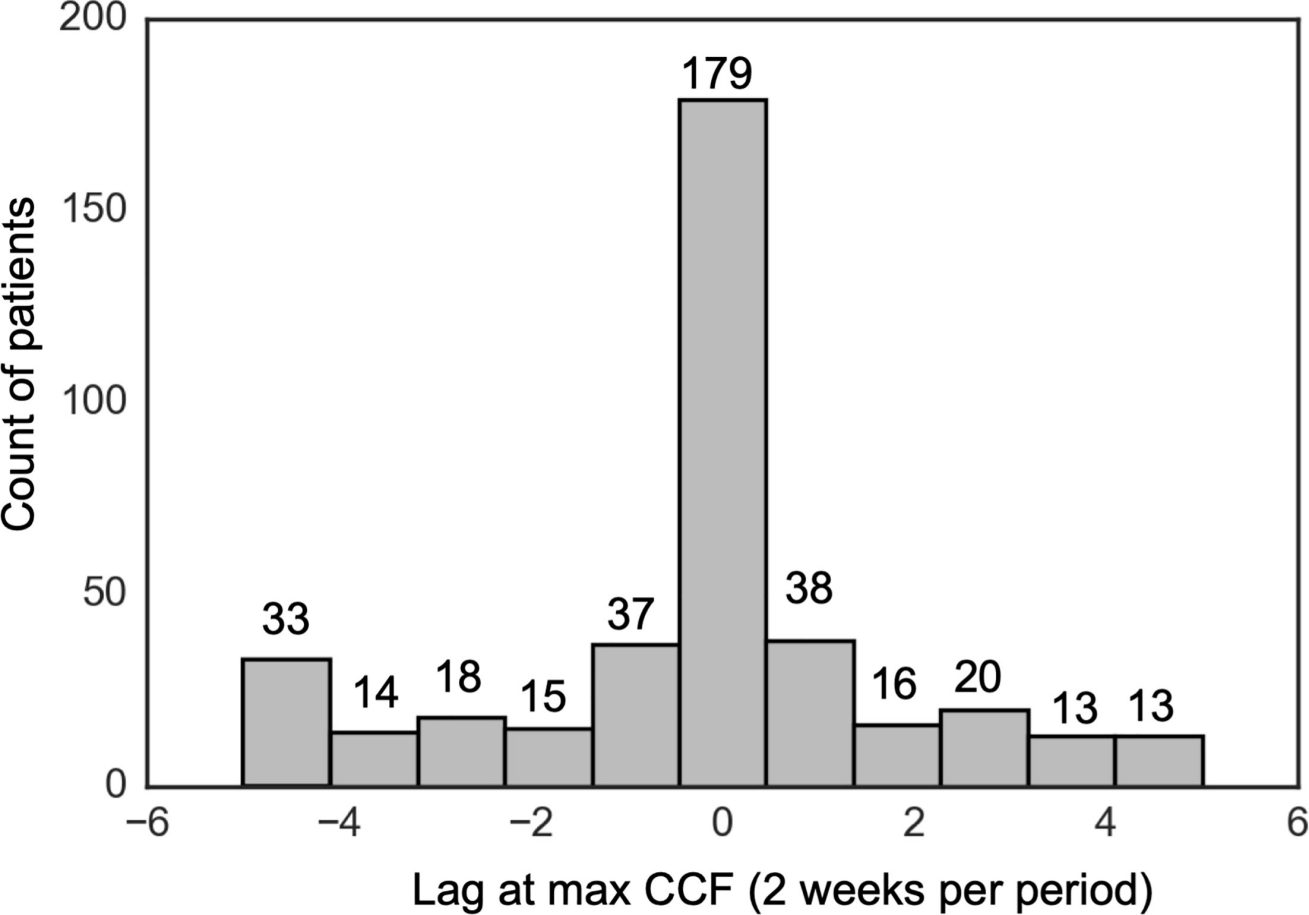
Histogram of the lag *k* at maximum CCF in the population, PHQ-8 vs Item 9. We excluded 214 patients with zero CCF between PHQ-8 and Item 9 for all lags between -5 and 5.

We then investigated the distribution of the lag at maximum CCF among the 394 patients with nonzero CCFs. Results are shown in Fig 2. For PHQ-8 vs Item 9, the majority of the patients have their PHQ-8 and Item 9 scores moving with the same trend with no lag; other patients have their PHQ-8 score leading Item 9 score with some time delay, or vice-versa; the period of delay is roughly uniformly distributed.

### PHQ-8 and Item 9 changing patterns

**Fig 3:**
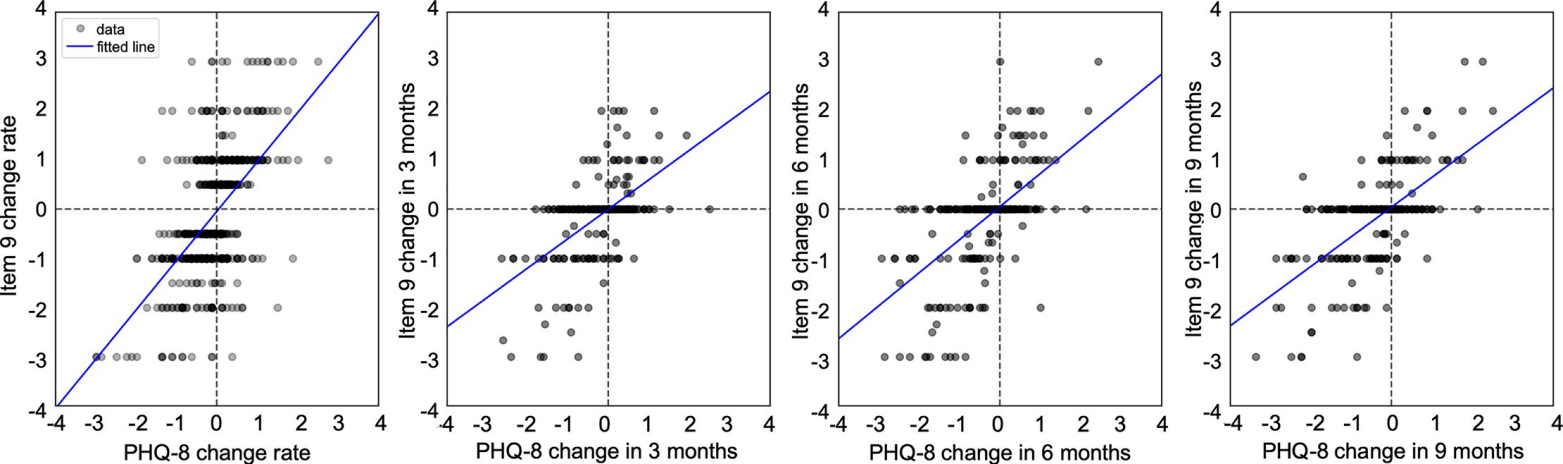
The association between the changes of PHQ-8 and Item 9 in both short term (less than 1 month) and long term (at 3, 6, 9 months). The solid lines are the result of linear regression.

The short-term association is indicated by the Spearman’s rank-order correlation calculated using the rate of change of the consecutive PHQ-8 and Item 9 values within 1 month. In Table 1, the result shows that the two scores have a positive monotonic relationship in the short term. We also conducted a linear regression using the same data, in which the result indicates a positive correlation (i.e. slope of the regression line is positive; see Fig 3). To examine the long-term effect, we computed the Spearman’s rank-order correlation between the changes of PHQ-8 and Item 9 at month 3, 6, 9 from month 0, separately. The results also show strong positive correlations in all three cases (see Table 1 and Fig 3). Therefore, we found that in majority of the cases in our EHR sample, patients’ PHQ-8 and Item 9 scores tend to change in the same direction.

**Table 1:** Spearman’s rank-order correlation and linear regression for PHQ-8 and Item 9.

		Spearman’s rank-order correlation (p value)	Slope of linear regression (R^2^ value)
Short-term (within 1 month)		0.52 (p<10^−4^)	1.00 (R^2^ = 0.31)
Long-term	3 months	0.53 (p<10^−4^)	0.60 (R^2^ = 0.30)
	6 months	0.57 (p<10^−4^)	0.67 (R^2^ = 0.34)
	9 months	0.56 (p<10^−4^)	0.60 (R^2^ = 0.39)

Next, we split the population of 396 patients (Item 9 not all-zeros) into four mutually exclusive and collectively exhaustive subgroups (see Table 2). We found that around 8% to 13% of the depression patients have experienced an increase in suicidal ideation during improvement of PHQ-8 scores (i.e. pattern b), based on different thresholds of defining changes in PHQ-8 scores. Therefore, the claim in [7] is partially supported in our EHR study sample. Next, we performed the chi-square test of homogeneity on these four subgroups (see Appendix A in S1 File), to test if they have significantly different distributions on certain categorical features, such as age, sex and mean of Charlson Comorbidity Index at a significance level of *α* = 0.05. We found no significant differences in distributions for any categories at all thresholds. However, we found that the distribution of age can be considered as different across the subgroups at a threshold of four units (a significance level of *α* = 0.06). Details of the categorical features and results of the chi-square test are listed in Appendix A in S1 File.

**Table 2:** Subgroup size for patients with various PHQ-8 and Item 9 changing patterns. Pattern (a): PHQ-8 increases and Item 9 decreases; Pattern (b): PHQ-8 decreases and Item 9 increases.

Threshold of PHQ-8 change	Number of patients in each subgroup			
	patients with pattern (a) only	patients with pattern (b) only	patients with both patterns	patients with no patterns
2	59 (14.9%)	53 (13.3%)	41 (10.4%)	243 (61.4%)
3	47 (11.9%)	41 (10.3%)	23 (5.8%)	285 (72.0%)
4	38 (9.6%)	31 (7.8%)	17 (4.3%)	310 (78.3%)

### Subtype discovery in depression trajectory patterns

Next, we discovered patterns in trajectories of depressive symptoms using an ANN. The inputs of the autoencoder are the fitted observations of the PHQ-8 and Item 9 records of each patient from GPR (input size *M* = 20, for 20 biweeks), which are further normalized to zero mean and unit variance. By performing cross validation in training the autoencoder, we found that the latent layer with very small size is not able to contain a sufficient variety of hidden features in terms of the shape of the hidden pattern. On the other hand, a very large latent layer results in large amount of repeated hidden features. We selected the number of latent nodes to be 25 (*H* = 25), based on both the results from cross-validation, and general rules of designing neural network structure found in literature, such as “the number of latent units no more than twice of the inputs” in [29]. The cross-validation also prevents the issue of overfitting.

**Fig 4:**
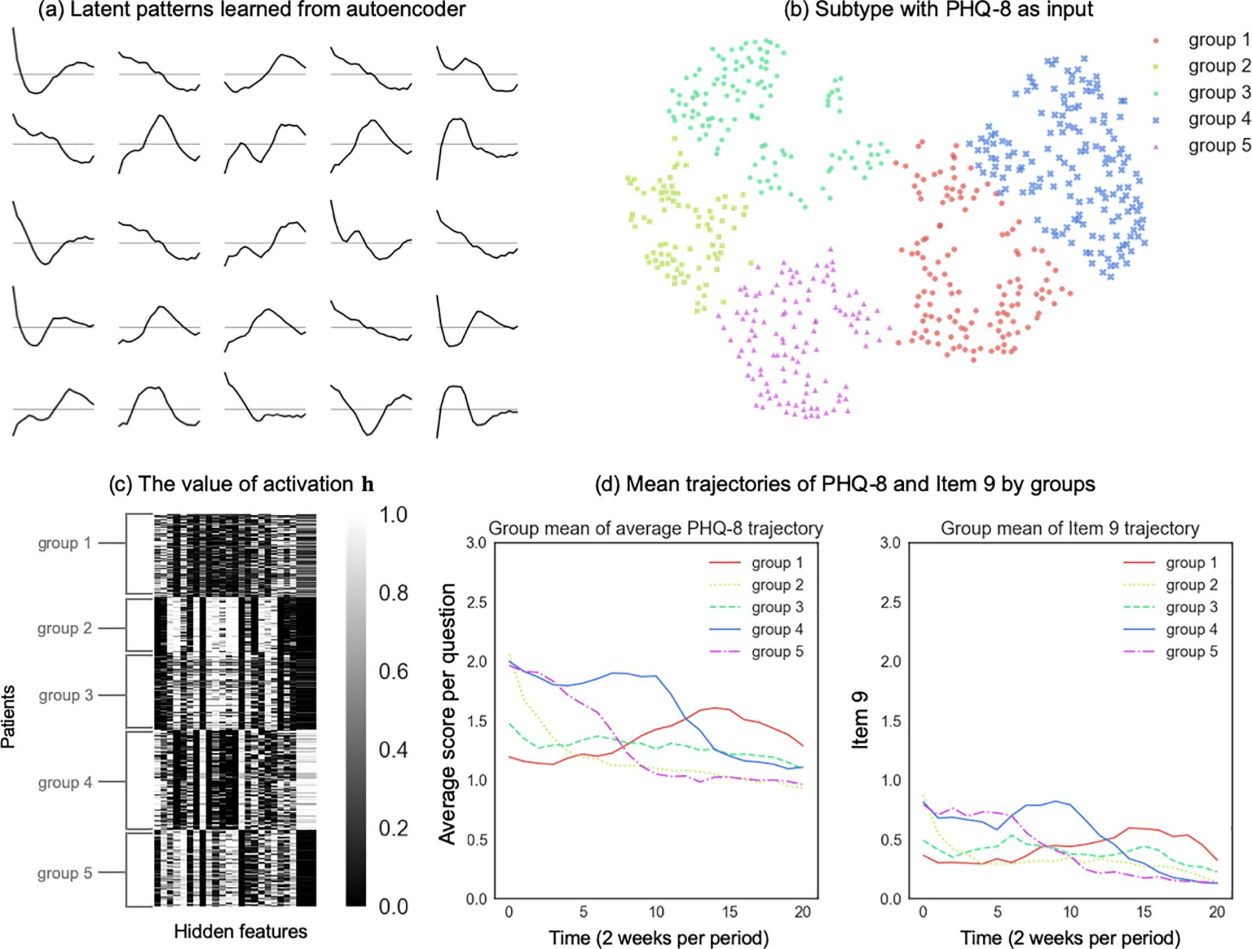
The subtype analysis result with hidden structures learned from the PHQ-8 trajectories. (a) Latent patterns learned from the PHQ-8 data. These patterns are visualized as the rows **W**_*i*_. In each panel, the x-axis is the time with a period of 2 weeks, and the y-axis represents the PHQ-8 scores of each basis trajectory. (b) Embed the activation **h** (25 dimensions) of each patient into 2-dimensional space with t-SNE and cluster them with the k-means algorithm in the 2-dimensional space. (c) The value of activation **h** on each latent pattern (25 columns) of each patient (610 rows) after reordering the rows by the clustering. (d) Mean trajectories of average PHQ-8 and Item 9 by groups, using the clustering results. One unit of time is two weeks. We used the average score of the first 8 questions in the PHQ to represent PHQ-8, which has the same range of 0 to 3 to Item 9.

We present the results using PHQ-8 trajectories as input in this section (results on Item 9 as input can be found in Appendix C in S1 File). The learned patterns of PHQ-8 are interpreted as a set of basis trajectories, which can be linearly combined to represent each patient’s trajectory. Fig 4(A) shows 25 latent patterns: many latent patterns indicate simple trend (e.g. the increasing trend, the decreasing trend, and the stable trend, etc.). Some latent patterns contain multiple trends, such as increasing first and then becoming stable, or increasing first and then decreasing. A small portion of the latent patterns has a periodic behavior within a short time.

Next, we investigated the similarity of each patient’s activation (**h**) feature on each latent pattern by embedding the activation in a two-dimensional space using t-Distributed Stochastic Neighbor Embedding (t-SNE) [30], which preserves clusters in the original data and reveals substructures within clusters. The activation of the population in the reduced dimensional space from t-SNE using PHQ-8 data as input is shown in Fig 4(B). We grouped patients into clusters by applying the k-means clustering algorithm on the transformed activation in 2-dimensional space, such that the clusters represent the latent structure. The clustering resulted in five subgroups. A heuristic way to validate the clustering result is to reorder the rows of the matrix of activation values (**h**) by grouping the patients within the same cluster. We plotted the value of the activation matrix after reordering by the clustering result learned; see Fig 4(C). It is evident that the features of activation in the same group are similar, and those in different groups have apparent dissimilarity.

Another approach to validate the clustering result is to show how distinguishable the mean trajectories are among different groups. The mean trajectory is the average measurement of the members in each group taken at each time point. Fig 4(D) shows the mean trajectories of PHQ-8 and Item 9 by the clustering result using the hidden patterns learned from the PHQ-8 trajectories. We observed that group 2, 4 and 5 had a trend of decreasing PHQ-8 over time, group 1 had a trend of PHQ-8 increasing first and then decreasing, and group 3 had a trend of relative stability. In Appendix C in S1 File, we provide the results of the mean trajectories by clustering using latent patterns learned from the Item 9 trajectories. The result was similar to the above, that the mean trajectories were more distinguishable among groups when using the same measurement as input. The five subgroups were similar between PHQ-8 and Item 9, which means patients in each group followed the same average patterns in their PHQ-8 and Item 9 trajectories (e.g. comparing the right and left panels of Fig 4(D)).

## Conclusions

This study described the longitudinal association between depression severity and suicidal ideation, and investigated the specific concern that suicidal ideation may increase during a period of depression improvement. We estimated the temporal correlation between two trajectories of depressive symptoms by first transforming depression records (PHQ-8 and Item 9) into continuous longitudinal trajectories, and then using the cross-correlation function. It is worthwhile to note that PHQ-8 and Item 9 have a strong temporal correlation; 45% of the patients with nonzero CCFs in our dataset have their PHQ-8 and Item 9 scores change in synchrony. The symmetric distribution of lag scores indicates the existence of patients in which their changes in suicidal ideation either precede or follow changes in overall depression severity. Our method provides useful insights to the practice of depression monitoring and suicide prevention; if we can determine a patient’s progression pattern using the cross-correlation analysis (i.e., the lag at the highest CCF between two trajectories), knowing the leading trajectory can provide strong evidence in the prediction of the following trajectory.

We used an ANN to discover the latent structure of the depressive symptom trajectories of a treatment population. The latent structure provides insights on the basic patterns of the depression progression. We further exploited this structure to classify patients into subtypes and displayed the mean trajectories of the clustered groups. We found five subtypes by the local patterns from the trajectories of the PHQ-8 scores. This result is similar to recent research on subtype identification in depression, including [20], [9], and [13]. Our results showed that the trend of a measurement (PHQ-8 or Item 9) is more distinguishable when the classification is based on the features of latent patterns learned from the trajectories of the same measurement, but the overall patterns are similar between PHQ-8 and Item 9 in the same group.

We note the following contributions. First, to the best of our knowledge, this paper is the first time-series study on the temporal correlation between PHQ-8 and Item 9 scores of the PHQ depression questionnaire using EHR data. It is different from the time-series analysis in [31] on daily changes in mindfulness, repetitive thinking, and depressive symptoms during a mindfulness-based treatment. Gunn et al. [9] has proposed that when the measurement of depressive symptoms is collected every three months, it is possible that some people may have experienced short-lived changes in their depression status between measurement periods. Our study has improved on existing literature by selecting 610 patients with more frequent records of PHQ-9 scores during a 40 weeks period. Second, we reached similar conclusions in the cross-correlation analysis using fitted trajectories, and changing-pattern analysis using unfitted raw depression scores; in the majority of patients in our sample, their PHQ-8 and Item 9 scores tend to change in the same direction. We also found that 8% to 13% of the patients have experienced an increase in suicidal ideation during improvement of PHQ-8 scores. This work is the first study to show evidence that a subgroup of depressive patients are at increased risk of suicide ideation during recovery using EHR data.

Our study is based on the assumption that thought of self-harm is an indicator to subsequent suicide attempt. There have been some debate on how significantly these two factors are associated. Two meta-analyses of longitudinal studies over the past decades concluded that the ability of self-injurious thought and behavior to predict suicidal attempt is weak [5,6]. However, Simon et al. [4] found that the Item 9 of the PHQ-9 is a strong predictor of suicide attempt, after adjustment for age, sex, treatment history, and overall depression severity, by using a EHR dataset of over 80,000 patients. There are several recent studies aim to predict suicide and examine suicidal behaviors using large-scale EHR data ranging from thousands to millions of patients [1,3,32,33]. For example, Simon et al. [3] designed a logistic regression model to predict suicide risk using EHR data of over 2.9 million patients. Ahmedani et al. [34] examined 19 physical health conditions on over 2,000 individuals who died by suicide and found that traumatic brain injury had the strongest association with increased suicide risk. These new results based on health records of large number of depressive patients have supported our assumption that suicidal ideation is an important risk factor to examine in order to prevent suicide attempts. We note that our study sample of 610 patients appears to be small in comparison, this is because we focused on a chronic depression treatment population with frequent observations to study their temporal patterns, which has much more restrictive data requirement than these large suicide risk prediction studies.

There are several promising future research directions with data containing detailed treatment information or a more comprehensive set of patients’ features. First, researchers have investigated the possibility that increases in suicidality may be related to the use of antidepressant medications [35]. Investigating the correlation between depression symptoms and suicide ideation trajectories by antidepressant types will shed more light on this issue. Second, examining depression symptom trajectories by specific treatment types would be illuminating in finding the difference between trajectories relative to the use of psychotherapy or antidepressant medication. Third, with larger datasets containing a richer set of patients’ features, we can discover emerging trends or pattern changes over time, or between different datasets using emerging pattern minding [36] or temporal pattern mining [37].

There are several limitations of this work. First, the Gaussian process regression is appropriate in the transformation of PHQ-8 scores which range from 0 to 24, and the variance is small for an individual patient. However, for sparse data like Item 9 which has the majority of measurements being zeroes, the GPR does not have a significant advantage compared to other interpolation methods like linear spline. Second, the results are based on a small patient cohort that are frequently monitored. Selection of patients with frequent visits may be biased toward those with more severe illness, but this is the patient group of greatest concern regarding worsening of suicidal ideation. All records rely on self-report and the patients in the dataset are mostly older adults (middle-aged and older) living in the U.S. western states. It may not be representative of young patients or those from different cultural background. Furthermore, our sample include only patients receiving treatment for depression, thus our findings may not be generalizable to people with unrecognized or untreated depression. Third, our data does not include ample information on demographic, education, income and other useful clinical factors to help us understand the findings. In addition, EHR data are sparse and irregular (e.g. we have no knowledge on whether there is a change of trend during a long gap between two consecutive PHQ records, which means that the changes calculated from a long period is not as reliable as the one from a shorter period). Fourth, the ANN implies a black-box model with limited interpretability on the latent representation **h** and row of **W**. Finally, the latent structure is a local pattern, which means we do not specify the starting point of this latent trajectory in the whole disease history. This will limit the application of this model to other chronic diseases for the following reason. Some clinical outcomes are periodic which are appropriate inputs to the latent structure learning model in this paper, while others are not periodic in which learning the local trend does not guarantee the trend in the long run. To apply this model to other disease applications, the periodicity of the clinical outcomes should be examined first.

In summary, we established a framework to extract subtypes of depression progression patterns from trajectories of depressive symptoms and examined the temporal relationship between PHQ-8 and Item 9 scores. This work contributes to the emerging field of personalized depression monitoring and suicide prevention by providing insights on analyzing temporal measurements to understand the association between different depressive symptoms. Future work can be extended to include additional measurements and clinical notes [33], such as examining temporal relationships between alcohol and drug use with depression symptoms trajectories.

## Supporting information

S1 File Appendix. (DOCX)
