# Supplementary material for: Machine learning discovery of longitudinal patterns of depression and suicidal ideation
Source: PLoS One. 2019 Sep 20;14(9):e0222665. doi: 10.1371/journal.pone.0222665 (PMC6754154; doi:10.1371/journal.pone.0222665)
Supplement: S1 File — (DOCX) [file pone.0222665.s001.docx]

**Machine Learning Discovery of Longitudinal Patterns of Depression and Suicidal Ideation**

Jue Gong, PhD^1^, Gregory E. Simon, MD, MPH^2,3^, Shan Liu, PhD^1*^

^1^Department of Industrial and Systems Engineering, University of Washington, Box 352650, Seattle, WA 98195, United States

^2^Kaiser Permanente Washington Health Research Institute, 1730 Minor Ave, Suite 1600, Seattle, WA 98101, United States

^3^Psychiatry and Behavioral Sciences, University of Washington, Box 356560, Seattle, WA 98195, United States

***Corresponding author**: Shan Liu, liushan@uw.edu

Mechanical Engineering Building, Room G6

Box 352650, University of Washington

Seattle, WA 98195

Office 206-543-7593. Fax 206-685-3072

**Appendix A. Details of Data Analyses**

**Summary of PHQ-9 data**

**Table A: Summary statistics of the 610 on-going treatment patients.**

| **Age count (%)** |  |
| --- | --- |
| 18-29 | 63 (10.3%) |
| 30-44 | 169 (27.7%) |
| 45-64 | 292 (47.9%) |
| 65+ | 86 (14.1%) |
| **Sex count (%)** |  |
| Female | 414 (67.9%) |
| Male | 196 (32.1%) |
| **Average PHQ-9** |  |
| Min | 0.10 |
| 25% Quantile | 8.20 |
| Median | 12.17 |
| 75% Quantile | 16.00 |
| Max | 25.22 |
| **Average PHQ-8** |  |
| Min | 0 |
| 25% Quantile | 8.01 |
| Median | 11.59 |
| 75% Quantile | 15.36 |
| Max | 23.88 |
| **Average Item 9** |  |
| Min | 0.00 |
| 25% Quantile | 0.00 |
| Median | 0.27 |
| 75% Quantile | 0.83 |
| Max | 2.75 |

**Chi-square analysis of homogeneity for PHQ-8 and Item 9 changing patterns**

We define the following categorical features

(1) Age: 4 levels: 18~29, 30~44, 45~64, 65+

(2) Sex: 2 levels: Male, Female

(3) Mean of Charlson Index: which is a measure of patients’ comorbid conditions, 4 levels

0: 0 ≤ mean Charlson Index < 0.5

1: 0.5 ≤ mean Charlson Index < 1

2: 1 ≤ mean Charlson Index < 2

3: 2 ≤ mean Charlson Index

Suppose the data were sampled from $r=4$ subgroups and the categorical feature has $c$ levels. The subgroups are

(1) patients with pattern (a) only

(2) patients with pattern (b) only

(3) patients with both patterns

(4) patients with no patterns

Recall from the main paper that the two patterns are (a) PHQ-8 increases and Item 9 decreases, and (b) PHQ-8 decreases and Item 9 increases (using unfitted observations in the EHR dataset). The categorical features are

(1) Age, with 4 levels

(2) Sex, with 2 levels

(3) Mean of Charlson Index, with 4 levels

At any specified level of the categorical variable, the null hypothesis states that each subgroup has the same proportion of observations. The degree of freedom is $\mathrm{DF}=\left( r-1 \right)\times\left( c-1 \right).$ The expected frequency counts are computed separately for each subgroup at each level of the categorical variable, $E_{r,c}=n_{r}\times n_{c}/n,$ where $E_{r,c}$ is the expected frequency count for subgroup $r$ at level $c$ of the categorical variable, $n_{r}$ is the total number of observations from subgroup $r$, $n_{c}$ is the total number of observations at level $c$, and $n$ is the total sample size. The test statistic is a chi-square random variable defined by

$$\chi^{2}=\sum_{i=1}^{c} \sum_{j=1}^{r} \frac{\left( O_{r,c}-E_{r,c} \right)^{2}}{E_{r,c}},$$

where $O_{r,c}$ is the observed frequency count in subgroup $r$ for level $c$ of the categorical variable, and $E_{r,c}$ is the expected frequency count in subgroup $r$ for level $c$ of the categorical variable. Finally, the p-value is defined as $p=P\left( x>\chi_{\mathrm{DF}}^{2} \right)$ [1].

Results of the Chi-square Test on Homogeneity for the four (mutually exclusive and collectively exhaustive) subgroups and for the four features are shown in Table B. The test is repeated for different threshold for defining PHQ-8 changes (threshold $d = 2, 3, 4$). We found no significant differences in distributions for any categories at all thresholds.

**Table B: The p-value of Chi-square Test on Homogeneity for various features.**

| Threshold for  PHQ-8 changes ($d$) | p-value of Chi-square Test on Homogeneity for features | | |
| --- | --- | --- | --- |
|  | Age | Sex | Mean of Charlson Index |
| 2 | 0.6049 | 0.8085 | 0.4035 |
| 3 | 0.3997 | 0.5672 | 0.1657 |
| 4 | 0.0570 | 0.6500 | 0.1345 |

**Appendix B. Details of methods**

**Gaussian Process Regression**

Gaussian process regression (GPR) is used to transform the sparse longitudinal data of PHQ records into a continuous curve. We follow the method and notation as defined in Lasko et al. [2]. The GPR model assumes that there is that the true depressive symptom trajectory (such as PHQ-8 and Item 9 scores) is represented as a latent source function $f(t)$. The observed depression sequence $y$ is considered as a set of samples taken from the source function, with observation noise. The probability of a given continuous function $f(t)$ in terms of an infinite-dimensional Gaussian process $\mathcal{G}\mathcal{P}$ is defined as

$$\begin{aligned} \Pr\left( f\left( t \right) \right)\mathcal{=GP}\left( m\left( t \right),C\left( t_{1},t_{2} \right) \right),\#\left( 1 \right) \end{aligned}$$

The mean function $m(t)$ is a function of time, and the covariance function $C\left( t_{1},t_{2} \right)$ is a function of the pair of times $t_{1}$ and $t_{2}$, which defines the dependence between two function values $f(t_{1})$ and $f(t_{2})$. The Gaussian process defined by $C$ represents a prior probability density over all possible source functions for the given trajectory. GPR produces a second Gaussian process that represents a posterior probability density given the prior and the observations in the trajectory.

Although the Gaussian process represents the probability density of the continuous function $f(t)$, the density $P(f(t_{i}))$ can be calculated at a finite set of times $t_{i}$. Given a vector of observations $\mathbf{y}^{0}\in\mathbb{R}^{n}$ made at times $\mathbf{t}^{0}\in\mathbb{R}^{n}$ , we can compute the posterior probability $\Pr(f\left( t \right)=y|\mathbf{y}^{0} ,\mathbf{t}^{0})$ that the true source function$f$ passes through the point $(t,y)$, which also represents the probability that a new measurement made at time $t$ would produce the value $y$. GPR assumes that at any time $t$, the posterior density is Gaussian,

$$\begin{aligned} \Pr\left( f\left( t \right)=y | \mathbf{y}^{0},\mathbf{t}^{0} \right)=\frac{1}{\sqrt{2\pi\hat{\sigma}^{2}}}\exp\left[ -\frac{\left( y-\hat{y} \right)^{2}}{2\hat{\sigma}^{2}} \right], \#\left( 2 \right) \end{aligned}$$

where $y=\mathbf{k}^{\top}\mathbf{K}^{-1}\mathbf{y}^{0}$ is the posterior mean value, $\hat{\sigma}^{2}=\kappa-\mathbf{k}^{\top}\mathbf{K}^{-1}\mathbf{k}$ is the posterior variance, $\mathbf{K}$ is a matrix with elements $\mathbf{K}_{ij}=C(\mathbf{t}_{i}^{0},\mathbf{t}_{j}^{0})$, $\mathbf{k}$ is a vector with elements $\mathbf{k}_{i}=C(\mathbf{t}_{i}^{0} ,t)$, and $\kappa=C(t,t)$ is a scalar. Equation (2) is used to compute the functions representing the best estimate $\hat{y}(t)$, the uncertainty in the estimate $\hat{\sigma}^{2}$, and the probability density $\Pr(f\left( t_{i} \right)=y|\mathbf{y}^{0},\mathbf{t}^{0})$over values of $y$, all calculated at times $t_{i}$. This is the goal of the transformation step. The estimate of $\hat{y}$ and $\hat{\sigma}$ is controlled with the rational quadratic function

$$\begin{aligned} C_{\mathrm{RQ}}\left( t_{1},t_{2} \right)=\sigma^{2}\exp\left[ 1+\frac{\left( t_{1}-t_{2} \right)^{2}}{2\alpha\tau^{2}} \right]^{-\alpha}. \#\left( 3 \right) \end{aligned}$$

We can tune the hyperparameters of covariance functions for an optimal fitting using the exact marginal likelihood of the hyperparameters [3]. The hyperparameters we used in this paper is listed in Table C.

**Table C:** The hyperparameters for Gaussian process regression to transform PHQ-8 records and Item 9 records.

| Measurement | $\sigma^{2}$ | $\tau$ | $\alpha$ |
| --- | --- | --- | --- |
| PHQ-8 | 100 | 0.25 | 0.12 |
| Item 9 | 100 | 0.50 | 0.10 |

**Training of Autoencoder**

We can train the parameters of the autoencoder by the following optimization problem

$$\begin{aligned} J=\min_{\mathbf{W},\mathbf{W}^{'},\mathbf{b},\mathbf{b}'} \sum_{j=1}^{N} \sum_{i=1}^{M} \left( \hat{m}_{i}^{j}-m_{i}^{j} \right)^{2}+\lambda\sum_{k,l} \left( W_{kl}^{2}+W_{lk}^{'2} \right)+\beta\sum_{i=1}^{H} D\left( \eta,\hat{\eta}_{i} \right), \#\left( 4 \right) \end{aligned}$$

The first term is the squared-error loss between the input data (the depressive symptom trajectory) and the reconstructed data (the set of basic trajectories), where the superscript $j$ denotes the $j$-th input trajectory (out of $N$ total inputs), and the subscript $i$ denotes the $i$-th data point of each input trajectory. The second term is the regularization term to ensure the elements of $\mathbf{W}$ are small. The third term is the sparsity term to make the activation $\mathbf{h}$ sparse (i.e. most elements are near zero), so that the input trajectory is represented by only a small number of reconstructed trajectories. The sparsity measure $\hat{\eta}_{i}$ is the average activation of the $i$-th hidden node.

$$\begin{aligned} \hat{\eta}_{i}=\frac{1}{N}\sum_{j=1}^{N} h_{i}^{j},\#\left( 5 \right) \end{aligned}$$

The function $D$ is the Kullback-Leibler divergence [4]

$$\begin{aligned} D\left( \eta,\hat{\eta} \right)=\eta\log\frac{\eta}{\hat{\eta}}+\left( 1-\eta\right)\log\frac{1-\eta}{1-\hat{\eta}} ,\#\left( 6 \right) \end{aligned}$$

that forces all $\hat{\eta}_{i}$ to be close to the sparsity target $\eta$, and $\lambda$ and $\beta$ are tuning parameters. This cost function (4) produces a sparse autoencoder [5].

**Appendix C. The results of Item 9 trajectories**

**Figure A:** The subtype analysis result with hidden structures learned from the Item 9 trajectories. (a) Latent patterns learned from the Item 9 data. These patterns are visualized as the rows $\mathbf{W}_{i}$. In each panel, the x-axis is the time with a period of 2 weeks, and the y-axis represents the PHQ-8 scores of each basis trajectory. (b) Embed the activation $\mathbf{h}$ (25 dimensions) of each patient into 2-dimensional space with t-SNE and cluster them with the k-means algorithm in the 2-dimensional space. (c) The value of activation $\mathbf{h}$ on each latent pattern (25 columns) of each patient (610 rows) after reordering the rows by the clustering. (d) Mean trajectories of average PHQ-8 and Item 9 by groups, using the clustering results. One unit of time is two weeks. We used the average score of the first 8 questions in the PHQ to represent PHQ-8, which has the same range of 0 to 3 to Item 9.

**Appendix D. Unsupervised classification with K-means algorithm**

We treat the activation $\mathbf{h}$ as the features of each patient for further analysis of subtype detection. The k-means algorithm divides a set of $N$ samples into $K$ disjoint clusters $C$, each described by the mean $\mu_{j}$ of the samples in the cluster. The means are commonly called the cluster “centroids”. The k-means algorithm aims to choose centroids that minimize the inertia, or within-cluster sum of squared criterion

$$\begin{aligned} \sum_{i=0}^{n} \min_{\mu_{j}\in C} \left( \left\| x_{j}-\mu_{i} \right\|^{2} \right), \# \end{aligned}$$

The optimal choice of $K$ should keep a balance between maximum compression of the data using a single cluster, and maximum accuracy by assigning each data point to its own cluster [6]. As the number of clusters increases, we choose $K$ as the number of clusters if the inertia decreases fast for number of clusters smaller than $K$, and decreases slowly for number of clusters greater than $K$[2, 5]. For the initialization of the centroids, we use the k-means++ algorithm that aims at that minimize the intra-class variance [7].

**Reference**

1. Test of Homogeneity, Chi-Square Test of homogeneity, chi-square. In: Kirch W, editor. Encyclopedia of Public Health. Dordrecht: Springer Netherlands; 2008. p. 1386-.

2. Lasko TA, Denny JC, Levy MA. Computational phenotype discovery using unsupervised feature learning over noisy, sparse, and irregular clinical data. PloS one. 2013;8(6):e66341.

3. Rasmussen CE. Gaussian processes for machine learning. 2006.

4. Cover TM, Thomas JA. Elements of information theory: John Wiley & Sons; 2012.

5. Goodfellow I, Bengio Y, Courville A. Deep learning: MIT Press; 2016.

6. Lloyd S. Least squares quantization in PCM. IEEE transactions on information theory. 1982;28(2):129-37.

7. Arthur D, Vassilvitskii S, editors. k-means++: The advantages of careful seeding. Proceedings of the eighteenth annual ACM-SIAM symposium on Discrete algorithms; 2007: Society for Industrial and Applied Mathematics.
